# Electric‐Field Control of Low Damping Constant in Epitaxial Co_2_FeSi/LiNbO_3_ Multiferroic Heterostructures

**DOI:** 10.1002/advs.202511250

**Published:** 2025-09-17

**Authors:** Shinya Yamada, Takamasa Usami, Sachio Komori, Yoshio Miura, Kazuto Yamanoi, Yukio Nozaki, Tomoyasu Taniyama, Kohei Hamaya

**Affiliations:** ^1^ Center for Spintronics Research Network Graduate School of Engineering Science The University of Osaka 1‐3 Machikaneyama Toyonaka Osaka 560‐8531 Japan; ^2^ Spintronics Research Network Division Institute for Open and Transdisciplinary Research Initiatives The University of Osaka 2‐1 Yamadaoka Suita Osaka 565‐0871 Japan; ^3^ Department of Physics Nagoya University Chikusa‐ku Nagoya Aichi 464‐8602 Japan; ^4^ Faculty of Electrical Engineering and Electronics Kyoto Institute of Technology Matsugasaki, Sakyo‐ku Kyoto 606‐8585 Japan; ^5^ Research Center for Magnetic and Spintronic Materials National Institute for Materials Science (NIMS) Tsukuba Ibaraki 305‐0047 Japan; ^6^ Department of Physics Keio University Yokohama Kanagawa 223‐8522 Japan; ^7^ Center for Spintronics Research Network Keio University Yokohama Kanagawa 223‐8522 Japan

**Keywords:** Heusler alloys, LiNbO_3_, magnetization dynamics, multiferroic heterostructures

## Abstract

To develop electric‐field control of magnetization dynamics in a magnetic material for magnonic devices with low‐energy power consumption operation, an epitaxial half‐metallic Co_2_FeSi/LiNbO_3_ multiferroic heterostructure is experimentally demonstrated. The epitaxial Co_2_FeSi/LiNbO_3_ multiferroic heterostructure shows a low damping constant (α) of ∼0.006 and the value of α is decreased to ∼0.004 by applying an electric field. This means that the magnetization dynamics in an epitaxial half‐metallic Co_2_FeSi layer can be controlled via the piezoelectric strain of LiNbO_3_ through the magnetoelastic coupling. This study leads to a way toward the realization of magnonic devices with low‐energy power consumption operation.

## Graphical abstract

The development of multiferroic heterostructures with a half‐metallic property is one of the key technologies for electric‐field operatable magnonic devices. In this study, Co_2_FeSi/LiNbO_3_ multiferroic heterostructures with a low damping constant are experimentally developed and an electric‐field modulation of its magnetization dynamics is demonstrated.

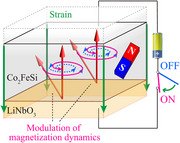

## 1 Introduction

Magnonics, which utilizes magnetization dynamics and spin wave propagation in a magnetic material as information carriers for processing, offers promising potentialities for novel computing and data processing approaches because of the absence of Joule heating.^[^ ^1^, ^2^, ^3^, ^4^, ^5^, ^6^ ^]^ For developing magnonic devices, lots of studies on magnetization dynamics and spin wave propagation in magnetic materials have been reported.^[^ ^7^, ^8^, ^9^, ^10^ ^]^ One of the conventional methods to excite magnetization dynamics and to manipulate spin wave propagation in a magnetic material is to apply an external microwave magnetic field. However, this method has been considered to be energy‐inefficient and impractical for device integration.^[^ ^11^ ^]^ From the viewpoint of low‐energy power consumption operation and high device integration, electric‐field control of magnetization dynamics and of spin wave propagation is desirable.^[^ ^12^, ^13^, ^14^, ^15^, ^16^ ^]^

To achieve this, the use of ferromagnetic/piezoelectric multiferroic heterostructures is one of the potential candidates because the magnetoelectric coupling can control the magnetic properties in the heterostructures above room temperature.^[^ ^12^, ^13^, ^14^, ^15^, ^16^, ^17^, ^18^, ^19^, ^20^ ^]^ Until now, electric‐field control of magnetization dynamics has been demonstrated in La_1−*x*_Sr_*x*_MnO_3_/Pb(Mg_1/3_Nb_2/3_)O_3_‐PbTiO_3_ (PMN‐PT) multiferroic heterostructures.^[^ ^14^, ^15^ ^]^ However, the value of damping constant (α) for the La_1−*x*_Sr_*x*_MnO_3_ film was ∼0.01,^[^ ^14^, ^15^ ^]^ insufficient for magnonic and spin‐wave device applications.

For long‐distance spin wave transport, the use of a magnetic material with a low α is essential. In addition, it is important to use a high‐quality epitaxial film because spin wave transport properties correlate with the crystallinity and the crystal orientation in the magnetic film.^[^ ^8^, ^10^ ^]^ Among a variety of magnetic materials, Co‐based Heusler alloys with the chemical formula Co_2_ *YZ*, where *Y* is a transition metal and *Z* is a main group element, are considered as candidate materials because many of them possess high spin polarization, low α, high Curie temperature, and so on.^[^ ^21^, ^22^, ^23^, ^24^, ^25^, ^26^, ^27^, ^28^ ^]^ In particular, Co_2_FeSi, one of the half‐metallic Co‐based Heusler alloys,^[^ ^29^ ^]^ has shown not only high room‐temperature spin polarization^[^ ^30^, ^31^ ^]^ and low α values (0.0023 – 0.0061)^[^ ^32^ ^]^ in single‐crystalline epitaxial films but also intriguing effects such as a giant converse magnetoelectric effect^[^ ^16^, ^18^, ^19^, ^20^, ^33^, ^34^ ^]^ and electric‐field control of anisotropic magnetoresistance^[^ ^35^ ^]^ in Co_2_FeSi/piezoelectric interfacial multiferroic systems.

**Figure 1:**
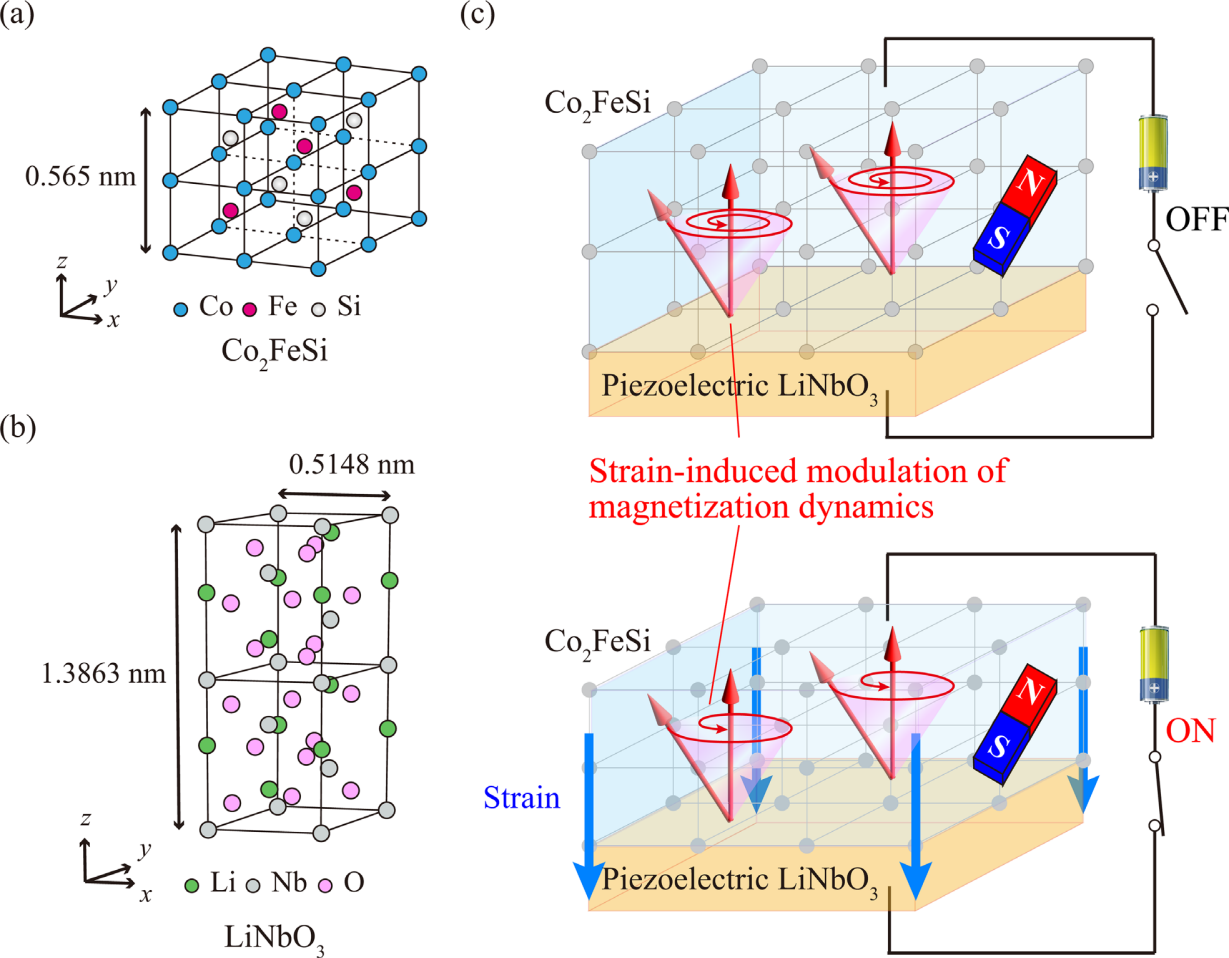
Crystal structures of a) *L*2_1_‐ordered Co_2_FeSi and b) LiNbO_3_. c) Schematic representation of the concept of this study. The lattice strain induced by piezoelectric LiNbO_3_ modulates the magnetization dynamics in Co_2_FeSi through the magnetoelastic coupling.

Until now, Co_2_ *YZ*/piezoelectric multiferroic heterostructures such as Co_2_ *YZ*/BaTiO_3_^[^ ^34^, ^35^ ^]^ and Co_2_ *YZ*/PMN‐PT^[^ ^16^, ^18^, ^19^, ^20^, ^33^, ^36^, ^37^, ^38^, ^39^ ^]^ have been intensively studied. However, piezoelectric LiNbO_3_, where surface acoustic waves (SAW) have been utilized to excite spin wave resonance in a magnetic material,^[^ ^40^ ^]^ has not thus far been explored as a growth substrate because the crystal structure of *L*2_1_‐ordered Co_2_ *YZ* (cubic structure) is completely different from LiNbO_3_ (trigonal ilmenite structure) and there is no matching between the lattice length and the symmetry, as shown in **Figure** **1**a,b.

In this research article, we demonstrate an epitaxial half‐metallic Co_2_FeSi/LiNbO_3_ multiferroic heterostructures with a low α. It is noted that the value of α in the epitaxial Co_2_FeSi layer is decreased from ∼0.006 to ∼0.004 by applying an electric field. This means that the magnetization dynamics in the epitaxial Co_2_FeSi layer can be controlled via the piezoelectric lattice strain of LiNbO_3_ through the magnetoelastic coupling. We discuss the possible origin of the strain‐induced modulation of α in Co_2_FeSi from the first‐principles calculation.

## 2 Results and Discussion

### 2.1 Epitaxial Co_2_FeSi/LiNbO_3_ Heterostructures

The concept of this study is schematically represented in Figure 1c. The lattice strain induced by the piezoelectric LiNbO_3_ modulates the magnetization dynamics in Co_2_FeSi through the magnetoelastic coupling. For the growth of an epitaxial Co_2_FeSi layer, LiNbO_3_ 128° Y‐cut (LN‐128Y) substrates were used.^[^ ^41^ ^]^ To solve the atomic arrangement mismatch problem between Co_2_FeSi and LN‐128Y (Figure 1a,b), we propose the use of Chromium (Cr) with a bcc crystal structure as an insertion layer.^[^ ^41^ ^]^ Cr has been widely used as a buffer layer for the growth of epitaxial Heusler‐alloy films on oxide substrates.^[^ ^26^, ^42^, ^43^, ^44^ ^]^ Although the crystal structure and the atomic arrangement between bcc‐Cr and LiNbO_3_ are also completely different, it was reported that molybdenum (Mo) with a bcc crystal structure was epitaxially grown on LN‐128Y.^[^ ^45^, ^46^ ^]^ Therefore, Cr can be used as an insertion layer for the growth of an epitaxial Co_2_FeSi layer on LN‐128Y.

Epitaxial Co_2_FeSi films were grown on LN‐128Y substrates by molecular beam epitaxy (MBE). The details of the growth process are described in the Experimental section (Supporting Information). Representative reflection high‐energy electron diffraction (RHEED) images during the growth are shown in Figure S1a–c (Supporting Information). After the LN‐128Y substrates were annealed at 500 °C for one hour in an MBE chamber, a 10‐nm‐thick Cr insertion layer is grown at a substrate temperature of 200 °C. As we conceived, clear streak patterns due to good 2D epitaxial growth are observed, as shown in Figure S1b (Supporting Information). Subsequently, a 30‐nm‐thick Co_2_FeSi layer is grown at a substrate temperature of 200 °C by co‐evaporating Co, Fe, and Si using Knudsen cells.^[^ ^47^, ^48^, ^49^ ^]^ After the growth of Co_2_FeSi, streak patterns due to epitaxial growth are observed, as shown in Figure S1a (Supporting Information). To confirm the effect of an insertion of the Cr layer, we also use Vanadium (V) with a bcc crystal structure as an insertion layer.^[^ ^50^ ^]^ As a result, although the surface flatness of a V insertion layer is not good compared with that of the Cr one (Figure S1e, Supporting Information), epitaxial growth of Co_2_FeSi is realized, as shown in Figure S1d (Supporting Information). By the way, if we directly grew Co_2_FeSi on LN‐128Y, only polycrystalline films could be obtained as shown in Figure S1f (Supporting Information). These results mean that the insertion of a bcc metal layer is essential to demonstrate epitaxial Co_2_FeSi/LN‐128Y heterostructures.

**Figure 2:**
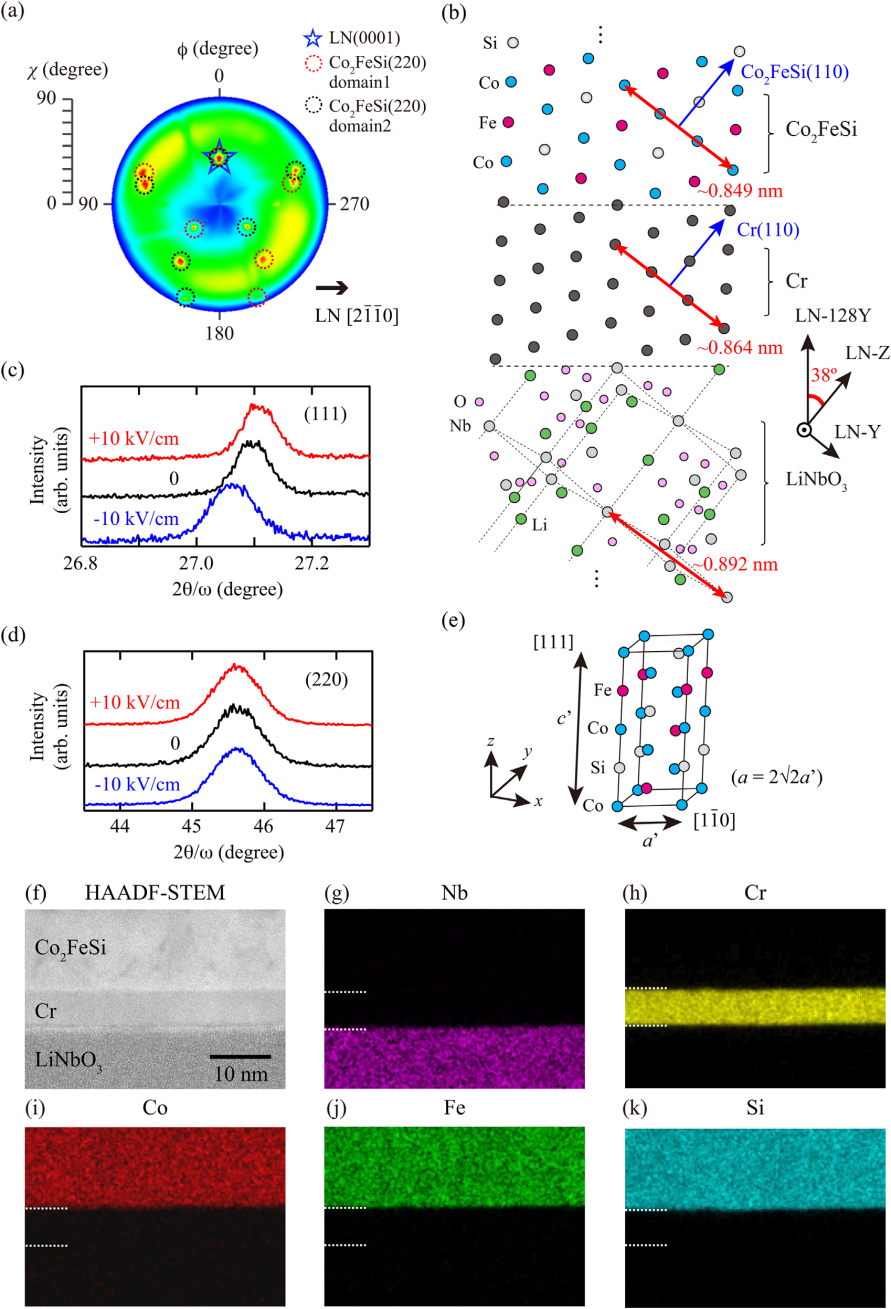
a) XRD pole figure of the Co_2_FeSi(220) plane for an epitaxial Co_2_FeSi/LN‐128Y heterostructure. b) Schematic of the cross‐sectional view of the epitaxial Co_2_FeSi/LN‐128Y multiferroic heterostructure. c,d) XRD 2θ‐ω scan measurements of the Co_2_FeSi(111) and Co_2_FeSi(220) planes for an epitaxial Co_2_FeSi/LN‐128Y multiferroic heterostructure under applying *E*. Schematic of the XRD 2θ‐ω scan measurements is shown in (e). f–k) HAADF‐STEM image and EDX mapping images for each element in an epitaxial Co_2_FeSi/LN‐128Y heterostructure.

To investigate the crystal orientation of the epitaxial Co_2_FeSi/LN‐128Y heterostructures, we performed x‐ray diffraction (XRD) measurements. **Figure** **2**a shows the pole figure of the Co_2_FeSi(220) plane for the epitaxial Co_2_FeSi/LN‐128Y heterostructure. Star and circle symbols represent the diffraction peaks of LN(0001) and Co_2_FeSi(220), respectively, and the LN(0001) peak is superimposed on the Co_2_FeSi(220) peaks (ϕ = 0°, χ = 38°). From this figure, it is revealed that the epitaxial Co_2_FeSi layer forms two crystal domains in the film plane, as shown in the circles of Figure 2a, and the Co_2_FeSi(110) plane is tilted at an angle of 38° from out‐of‐plane direction, as shown in a blue arrow of Figure 2b. This unique epitaxial relationship is the same as in the reported previous studies of epitaxial bcc‐Mo/LN‐128Y heterostructures.^[^ ^45^, ^46^ ^]^ As shown in the red arrows, although the mismatch between Co_2_FeSi (∼0.849 nm) and LiNbO_3_ (∼0.892 nm) is ∼5 %, the Cr insertion layer decreases the mismatch between Co_2_FeSi and LiNbO_3_. As a result, epitaxial growth of Co_2_FeSi on LN‐128Y is realized through the Cr insertion layer, as illustrated in Figure 2b.

To examine the effect of an electric field (*E*) on the lattice strain in the Co_2_FeSi layer, we also performed XRD measurements by applying *E*. Figure 2c,d shows the XRD 2θ‐ω scan measurements of the Co_2_FeSi(111) and (220) planes for the epitaxial Co_2_FeSi layer at *E* = −10 kV/cm (blue), 0 (black), and +10 kV/cm (red). To roughly confirm whether piezoelectric distortion propagated by external electric fields, electric fields were applied at only three points: −10 kV/cm, 0, and +10 kV/cm. From Figure 2a,b, there are no Miller indices corresponding to the vertical and horizontal directions of the epitaxial Co_2_FeSi layer, therefore, we assumed that the [111] and [1$\bar{1}$0] directions of Co_2_FeSi are the vertical and horizontal directions of the epitaxial Co_2_FeSi layer, as illustrated in Figure 2e. In Figure 2c, the Co_2_FeSi(111) peak due to the formation of the *L*2_1_‐ordered structure can be seen. By sweeping *E* from −10 to +10 kV/cm, diffraction peaks of the Co_2_FeSi(111) plane move toward higher angles. This indicates that the compressive (tensile) strain is applied along the out‐of‐plane direction by applying a positive (negative) *E*. For the (220) plane, on the other hand, we can see no peak shift before and after applying *E*. That is, while the in‐plane lattice constant (*a*′) does not change with respect to *E*, the out‐of‐plane lattice strain constant (*c*′) is varied by applying *E* and positive (negative) *E* induces the out‐of‐plane compressive (tensile) strain in the epitaxial Co_2_FeSi layer. This feature can be qualitatively understood from the piezoelectric lattice strain of LiNbO_3_.

We further carried out structural characterizations by cross‐sectional high‐angle annular dark‐field scanning transmission electron microscopy (HAADF‐STEM) and energy dispersive x‐ray spectroscopy (EDX) measurements. Figure 2f–k shows a HAADF‐STEM image and EDX mapping ones for each element of an epitaxial Co_2_FeSi/LN‐128Y heterostructure. A Cr layer with a flat surface is grown on an LN‐128Y substrate (Figure 2f). Because of the low‐temperature growth of 200 °C, atomic interdiffusion between the Cr insertion layer and the LN‐128Y substrate is suppressed (Figure 2g,h). On top of the flat Cr layer, a Co_2_FeSi layer is grown with suppressed atomic interdiffusion (Figure 2h‐k). These results indicate that our low‐temperature MBE techniques enable us to demonstrate an epitaxial Co_2_FeSi/LN‐128Y heterostructure without degrading piezoelectric properties of LiNbO_3_.

We also measured magnetic properties of the epitaxial Co_2_FeSi/LN‐128Y heterostructure. A field‐dependent magnetization (*M*‐*H* curve) at 300 K for an epitaxial Co_2_FeSi/LN‐128Y heterostructure in a high magnetic field region is shown in Figure S2a (Supporting Information), where an external magnetic field was applied was applied with an angle of 90° with respect to the LN[2$\bar{11}$0] direction (a red arrow in the inset figure). The saturation magnetization is ∼1235 emu/cc, almost equivalent to that for bulk^[^ ^29^ ^]^ and thin‐film^[^ ^47^, ^48^, ^51^ ^]^ samples reported previously. In‐plane normalized *M*‐*H* curves in a low magnetic field region are also shown in Figure S2b (Supporting Information). The epitaxial Co_2_FeSi/LN‐128Y heterostructure exhibits a strong in‐plane uniaxial magnetic anisotropy with an easy axis of the perpendicular to the LN[2$\bar{11}$0] direction. We speculate that the in‐plane uniaxial magnetic anisotropy is derived from the lattice strain induced by the change in the crystal structure from the substrate.^[^ ^52^, ^53^, ^54^, ^55^, ^56^ ^]^ From these structural characterizations and magnetic properties, we regard that epitaxial Co_2_FeSi/LN‐128Y multiferroic heterostructures are successfully grown by MBE techniques.

### 2.2 Magnetization Dynamics

**Figure 3:**
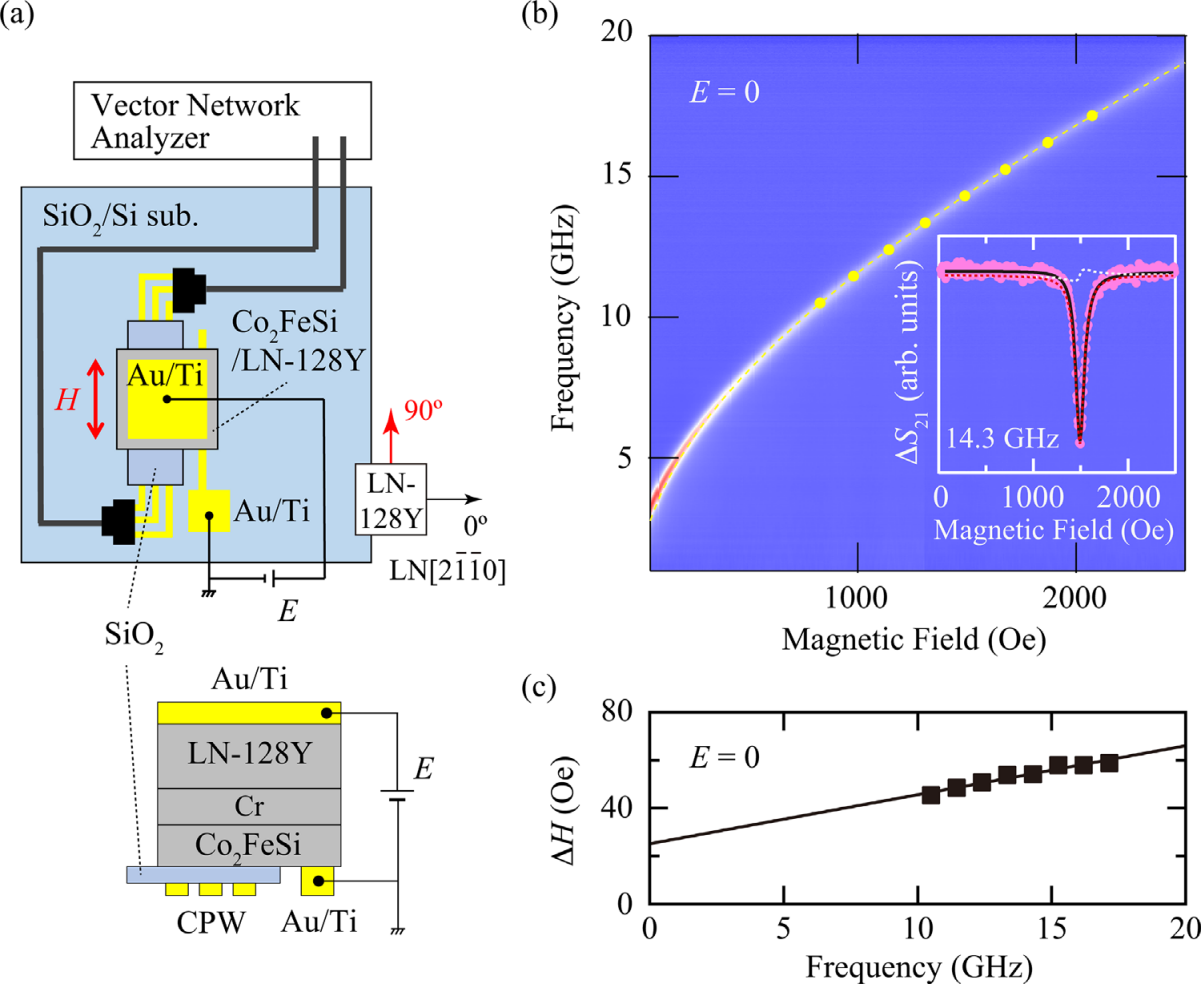
a) Top view of the experimental setup for FMR measurements. b) FMR spectra map for an epitaxial Co_2_FeSi/LN‐128Y multiferroic heterostructure at *E* = 0, where the external static magnetic fields were applied along the magnetic easy axis of the Co_2_FeSi layer (at an angle of 90° with respect to the LN[2$\bar{11}$0] direction, red arrow in the figure). A representative FMR spectrum is shown in the inset. c) Frequency dependence of Δ*H* at *E* = 0.

We measured the magnetization dynamics of Co_2_FeSi/LN‐128Y multiferroic heterostructures. The experimental set up for ferromagnetic resonance (FMR) measurements is shown in **Figure** **3**a. The details of the measurements are described in the Experimental Section (Supporting Information). Figure 3b shows the FMR spectra map for the epitaxial Co_2_FeSi/LN‐128Y multiferroic heterostructure at *E* = 0, where the external static magnetic fields were applied along the magnetic easy axis of the epitaxial Co_2_FeSi layer (a red arrow in Figure 3a). A representative FMR spectrum is shown in the inset. A clear FMR absorption with a small line‐width is observed. The FMR spectra are fitted with the following equation, which consists of symmetric and antisymmetric Lorentzians,^[^ ^57^ ^]^

(1) $$\begin{array}{c} \Delta S_{21}=C_{s}\frac{{\left(\Delta H\right)}^{2}}{{\left(\Delta H\right)}^{2}+{\left(H-H_{\mathrm{res}}\right)}^{2}}+C_{\mathrm{as}}\frac{\Delta H(H-H_{\mathrm{res}})}{{\left(\Delta H\right)}^{2}+{\left(H-H_{\mathrm{res}}\right)}^{2}}+B, \end{array}$$

where *C*_s_ and *C*_as_ are the parameters representing the amplitude of the symmetric and antisymmetric Lorentzians, *B* is the parameter representing the offset, Δ*H* is the half‐width at half maximum, and *H*_res_ is the resonant field. A fitting curve is shown as the solid black curve in the inset of Figure 3(b). Here since spin wave excitation with a wavelength near the line width of the coplaner waveguide (CPW) antenna is not necessary to be considered, the dispersion relationship between the resonant frequency (*f*_res_) and *H*_res_ is fitted using Kittel's equation,^[^ ^58^, ^59^ ^]^

(2) $$\begin{array}{c} f_{\mathrm{res}}=\left(\frac{1}{2\pi }\right)\left(\frac{ge}{2m_{0}c}\right)\sqrt{\left(H_{\mathrm{res}}+H_{a}\right)\left(H_{\mathrm{res}}+H_{a}+4\pi M_{\mathrm{eff}}\right)}, \end{array}$$

where, *g* is the *g* factor (= 2),^[^ ^60^ ^]^ *e* is the electron charge, *m*_0_ is the electron rest mass, *c* is the velocity of light, *H*_a_ is the anisotropic field, and *M*_eff_ is the effective magnetization. Figure 3c shows the extracted values of Δ*H* versus *f*_res_ for the epitaxial Co_2_FeSi/LN‐128Y multiferroic heterostructure. A clear linear relation between Δ*H* and *f*_res_ can be seen. From the frequency dependence of Δ*H* and the following equation,^[^ ^61^, ^62^ ^]^

(3) $$\Delta H=\Delta H_{0}+\frac{2\pi \alpha f_{\mathrm{res}}}{\gamma },$$

where γ is the gyromagnetic ratio and Δ*H*_0_ is the inhomogeneous broadening, the value of α is estimated to be ∼0.006, comparable to those for highly‐ordered epitaxial Co‐based Heusler‐alloy films.^[^ ^32^, ^63^, ^64^, ^65^, ^66^, ^67^ ^]^ Although the α value of ∼0.006 is small, the influence of the presence of two crystal domains in the epitaxial Co_2_FeSi layer on α needs to be considered because the epitaxial Co_2_FeSi layer is an imperfect single‐crystalline film. These results show that epitaxial half‐metallic Co_2_FeSi/LN‐128Y multiferroic heterostructures with a low α are obtained.

### 2.3 Effect of Strain on Damping Constant

**Figure 4:**
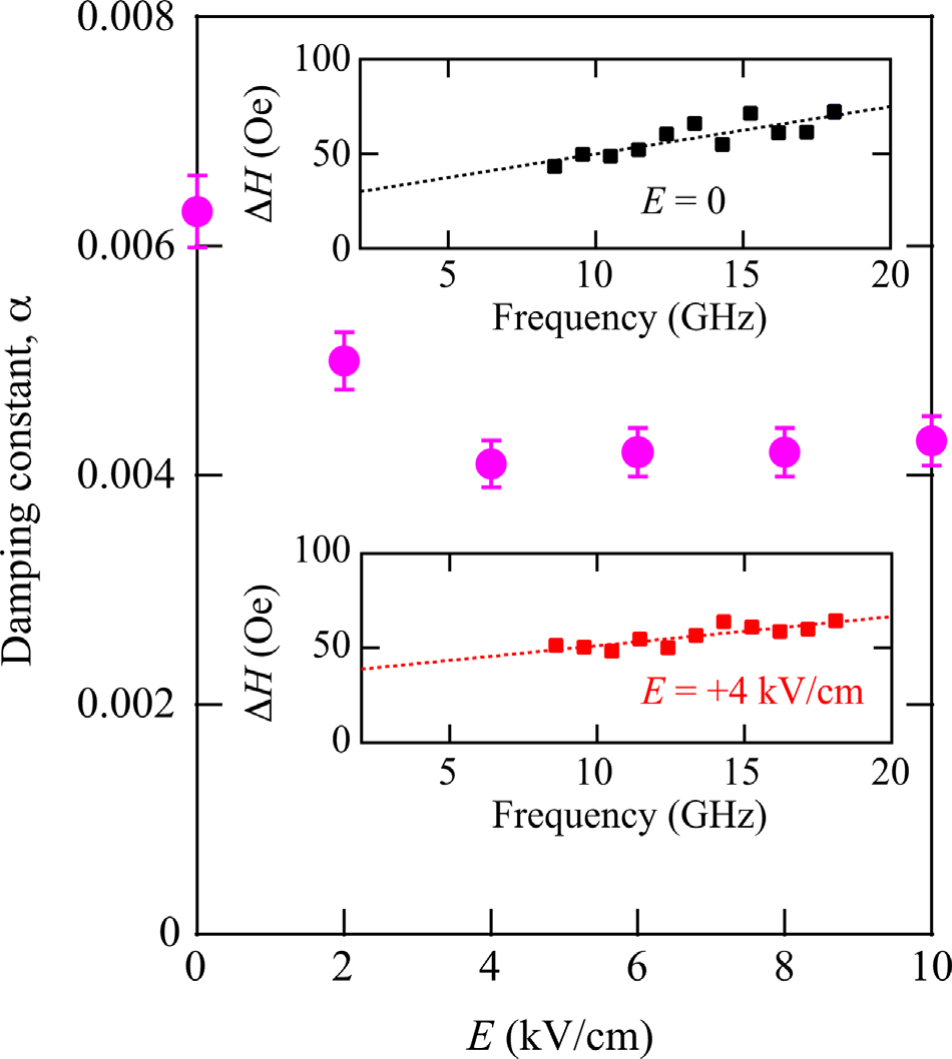
α versus *E* for an epitaxial Co_2_FeSi/LN‐128Y multiferroic heterostructure. The inset graphs show frequency dependence of Δ*H* at *E* = 0 (upper, black) and +4 kV/cm (lower, red).

We investigated the effect of *E* on the magnetization dynamics of an epitaxial Co_2_FeSi/LN‐128Y multiferroic heterostructure. Using the experimental setup shown in Figure 3a, we performed FMR measurements under applying *E*. For each measurement, we analyzed the data by following the same procedure as described above. **Figure** **4** shows α versus *E* for the epitaxial Co_2_FeSi/LN‐128Y multiferroic heterostructure. The inset graphs show the representative *f* dependence of Δ*H*. The value of α is decreased from ∼0.006 to ∼0.004 by applying positive *E*. This means that the magnetization dynamics in the epitaxial Co_2_FeSi layer can be modulated by applying *E*. We also notice an increase in Δ*H*_0_ applying *E* (inset figures), which is probably due to the magnetic inhomogeneity in the ferromagnetic domains induced by the lattice distortion from the piezoelectric LN‐128Y substrates. Although the electric‐field modulation of α was reported in La_1−*x*_Sr_*x*_MnO_3_/PMN‐PT multiferroic heterostructures, the modulation of α was in the order of ∼10^−2^.^[^ ^14^, ^15^ ^]^ For the epitaxial Co_2_FeSi/LN‐128Y multiferroic heterostructure, on the other hand, the magnitude of α is modulated in the order of 10^−3^. The Curie temperature of Co_2_FeSi is 1100 K,^[^ ^29^ ^]^ much higher than that of La_1−*x*_Sr_*x*_MnO_3_ (∼370 K),^[^ ^68^, ^69^ ^]^ and Co_2_FeSi has exhibited specific features in multiferroic heterostructures.^[^ ^16^, ^18^, ^19^, ^20^, ^33^, ^34^, ^35^ ^]^ Therefore, it is expected that the epitaxial Co_2_FeSi/LN‐128Y multiferroic heterostructures with a low α present a potential of the application for electric‐field‐controllable magnonic devices.

**Figure 5:**
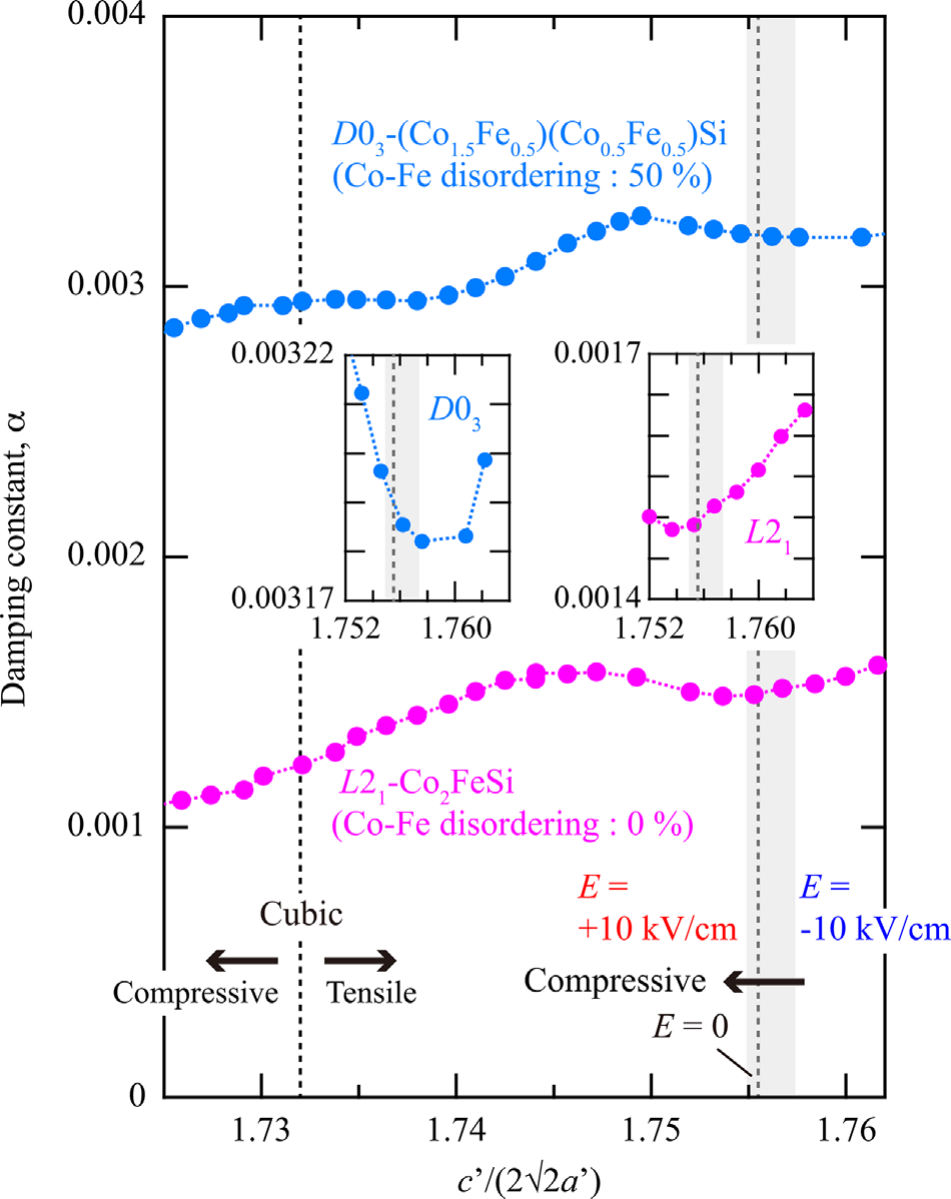
Theoretical values of α versus lattice strain, *c*′/(2$\sqrt{2}a^{\prime }$), for Co_2_FeSi with the Co–Fe disordering of 0 % (*L*2_1_‐Co_2_FeSi, magenta plots) and 50 % (*D*0_3_‐(Co_1.5_Fe_0.5_)(Co_0.5_Fe_0.5_)Si, cyan plots). Gray area is the experimental region of *c*′/(2$\sqrt{2}a^{\prime }$) calculated from the results in Figure 2c,d. The inset graphs show the enlarged views near *c*′/(2$\sqrt{2}a^{\prime }$) of 1.756 for *L*2_1_‐Co_2_FeSi (right, magenta plots) and *D*0_3_‐(Co_1.5_Fe_0.5_)(Co_0.5_Fe_0.5_)Si (left, cyan plots).

To understand the effect of the lattice strain on α, we performed the first‐principles calculations of α for Co_2_FeSi versus the lattice strain. The details of the first‐principles calculations are described in the Experimental Section (Supporting Information). As mentioned in the Section 2.1, there are no Miller indices corresponding to the vertical and horizontal directions of the epitaxial Co_2_FeSi layer, we assume that the out‐of‐plane of the Co_2_FeSi layer is defined as the Co_2_FeSi[111] direction, as shown in Figure 2e. Since *D*0_3_‐type disordering can easily occur in Co_2_FeSi even for the low‐temperature growth,^[^ ^51^ ^]^ we also considered the effect of structural disordering on α. **Figure** **5** displays the theoretical results of α versus lattice strain, *c*′/(2$\sqrt{2}a^{\prime }$), for Co_2_FeSi without the Co–Fe disordering (*L*2_1_‐Co_2_FeSi, magenta) and with the 50% Co–Fe disordering (*D*0_3_‐(Co_1.5_Fe_0.5_)(Co_0.5_Fe_0.5_)Si, cyan). The dotted magenta line at *c*′/(2$\sqrt{2}a^{\prime }$) of 1.732 is the case of cubic‐Co_2_FeSi. There is a large discrepancy in the magnitude of α between experiment and theory since the theoretical calculations do not include imperfection of the crystal and magnetic structures. As shown in Figure 2a, the epitaxial Co_2_FeSi layer contains two crystal domains in the film plane, which can become one of the possible reasons of the discrepancy in α between experiment and theory. The presence of some structural disorder and lattice strain in the epitaxial Co_2_FeSi layer also affects the discrepancy in α. Although the Cr insertion layer is antiferromagnetic, exchange bias cannot be observed from the low‐field *M*‐*H* curves in Figure S2b (Supporting Information). In addition, when the thickness of the Co_2_FeSi layer was decreased from 30 to 10 nm without changing the thickness of the Cr layer, the value of α was also ∼0.006 and exchange bias could not be observed from low‐field *M*‐*H* curves (not shown here). From these, we can judge that the influence of the exchange coupling between Co_2_FeSi and Cr is negligible and the mechanism of the modulation of α observed here is derived predominantly from the strain effect. Therefore, we compare the qualitative behavior of α with respect to *c*′/(2$\sqrt{2}a^{\prime }$) between experiment and theory.

The theoretical value of α for cubic *D*0_3_‐(Co_1.5_Fe_0.5_)(Co_0.5_Fe_0.5_)Si is larger than cubic *L*2_1_‐Co_2_FeSi because of the increase in the total DOS in the minority spin band at the Fermi level (*E*_F_), as shown in Figure S3a–d (Supporting Information). According to the theoretical model proposed by Kamberský^[^ ^70^ ^]^ and Gilmore *et al.*,^[^ ^71^ ^]^ it is predicted that the spin‐flip scattering can be strongly suppressed in half‐metallic materials because only a majority spin band exists at *E*_F_.^[^ ^72^ ^]^ As the total DOS in the minority spin band at *E*_F_ decreases, the spin‐flip scattering is suppressed, leading to a decrease in α. The theoretical α values near *c*′/(2$\sqrt{2}a^{\prime }$) of 1.732 (cubic‐Co_2_FeSi) tend to be small when the compressive strain is applied for both *L*2_1_‐Co_2_FeSi and *D*0_3_‐(Co_1.5_Fe_0.5_)(Co_0.5_Fe_0.5_)Si. This indicates that theoretical studies also suggest the value of α in the epitaxial Co_2_FeSi layer can be controlled by the lattice strain in piezoelectric LiNbO_3_ through the magnetoelastic coupling. From the XRD data in Figure 2c,d, the value of *c*′/(2$\sqrt{2}a^{\prime }$) was estimated to be ∼1.756 at *E* = 0 (a gray dotted line in Figure 5), indicating that the out‐of‐plane tensile strain is applied before applying *E*, probably caused by the crystal structural difference between Co_2_FeSi (and Cr) and LiNbO_3_. Focusing on the data near *c*′/(2$\sqrt{2}a^{\prime }$) of 1.756 (a gray area in Figure 5), the theoretical values of α for *L*2_1_‐Co_2_FeSi tend to decrease by applying the compressive strain (right inset figure) while those for *D*0_3_‐(Co_1.5_Fe_0.5_)(Co_0.5_Fe_0.5_)Si increase (left inset figure), therefore our experimental data are qualitatively consistent with the behavior of *L*2_1_‐Co_2_FeSi. Thus, we roughly judged that our epitaxial Co_2_FeSi layer contains relatively little *D*0_3_‐type disorder. Considering the fact that the values of the in‐plane lattice constant do not change by *E* (Figure 2d), we speculate that the application of the out‐of‐plane compressive strain to the epitaxial Co_2_FeSi layer induces the structural change toward the cubic‐Co_2_FeSi, leading to the decrease in α. At present, since only a qualitative interpretation of the phenomenon observed here is presented, further consideration will be needed to understand the modulation mechanism in Co_2_FeSi/LiNbO_3_ multiferroic systems in details.

Finally, we comment on the superiority of this study. Contrary to Co_2_FeSi/PMN‐PT^[^ ^16^, ^18^, ^19^, ^20^, ^33^ ^]^ and Co_2_FeSi/BaTiO_3_^[^ ^34^, ^35^ ^]^ multiferroic heterostructures, the epitaxial Co_2_FeSi/LiNbO_3_ multiferroic heterostructures are not influenced by ferroelectric domain structures in the piezoelectric LiNbO_3_ substrate because LiNbO_3_ has a uniform single ferroelectric domain structure. This is one of the important points in terms of controllability of magnetization dynamics. In addition, if spin wave generation by SAW on piezoelectric LiNbO_3_ substrates^[^ ^40^, ^73^, ^74^ ^]^ was implemented in our epitaxial Co_2_FeSi/LiNbO_3_ multiferroic heterostructures, electric‐field generation of spin waves could be demonstrated in the Co_2_FeSi/LN‐128Y multiferroic heterostructures. Furthermore, if antiferromagnetic materials were utilized in this multiferroic heterostructure, magnetization dynamics of antiferromagnetic materials could also be modulated via the strain‐induced variation of the density of states near the Fermi level.^[^ ^75^, ^76^, ^77^ ^]^ Therefore, we expect that the epitaxial half‐metallic Co_2_FeSi/LN‐128Y multiferroic heterostructures developed here lead to a step toward the realization of electric‐field‐operatable magnonic devices.

## 3 Conclusion

We have demonstrated epitaxial half‐metallic Co_2_FeSi/LiNbO_3_ multiferroic heterostructures by introducing a bcc‐Cr insertion layer. The epitaxial *L*2_1_‐Co_2_FeSi/LiNbO_3_ heterostructures showed a high saturation magnetization almost equivalent to theoretical values reported previously and a low damping constant of ∼0.006, indicating a half‐metallic nature. The values of α were modulated from ∼0.006 to ∼0.004 by applying an electric field, qualitatively explained in terms of the strain‐induced variation of the density of states near the Fermi level. This study leads to a step toward the realization of electric‐field‐operatable magnonic devices.

## Conflict of Interest

The authors declare no conflict of interest.

## Supporting information

Supporting Information

## Data Availability

The data that support the findings of this study are available from the corresponding author upon reasonable request.
