# Supplementary material for: Electric‐Field Control of Low Damping Constant in Epitaxial Co2FeSi/LiNbO3 Multiferroic Heterostructures
Source: Adv Sci (Weinh). 2025 Sep 17;12(41):e11250. doi: 10.1002/advs.202511250 (PMC12591117; doi:10.1002/advs.202511250)
Supplement: Supplementary file 1 — Supporting Information [file ADVS-12-e11250-s001.pdf]

Supporting Information: Electric-field Control of Low Damping Constant in Epitaxial $Co_2FeSi/LiNbO_3$ Multiferroic Heterostructures

Shinya Yamada Takamasa Usami Sachio Komori Yoshio Miura* Kazuto Yamanoi Yukio Nozaki Tomoyasu Taniyama Kohei Hamaya**

Prof. Dr. S. Yamada
Center for Spintronics Research Network, Graduate School of Engineering Science, The University of Osaka, 1-3 Machikaneyama, Toyonaka, Osaka 560-8531, Japan
Spintronics Research Network Division, Institute for Open and Transdisciplinary Research Initiatives, The University of Osaka, 2-1 Yamadaoka, Suita, Osaka 565-0871, Japan
E-mail: yamada.shinya.es@osaka-u.ac.jp

Dr. T. Usami
Center for Spintronics Research Network, Graduate School of Engineering Science, The University of Osaka, 1-3 Machikaneyama, Toyonaka, Osaka 560-8531, Japan
Spintronics Research Network Division, Institute for Open and Transdisciplinary Research Initiatives, The University of Osaka, 2-1 Yamadaoka, Suita, Osaka 565-0871, Japan

Dr. S. Komori
Department of Physics, Nagoya University, Chikusa-ku, Nagoya, Aichi 464-8602, Japan

Prof. Dr. Y. Miura
Faculty of Electrical Engineering and Electronics, Kyoto Institute of Technology, Matsugasaki, Sakyo-ku, Kyoto, 606-8585, Japan
Research Center for Magnetic and Spintronic Materials, National Institute for Materials Science (NIMS), Tsukuba, Ibaraki 305-0047, Japan
Center for Spintronics Research Network, Graduate School of Engineering Science, The University of Osaka, 1-3 Machikaneyama, Toyonaka, Osaka 560-8531, Japan
E-mail: miura@kit.ac.jp

Dr. K. Yamanoi
Department of Physics, Keio University, Yokohama, Kanagawa 223-8522, Japan
Center for Spintronics Research Network, Keio University, Yokohama, Kanagawa 223-8522, Japan

Prof. Dr. Y. Nozaki
Department of Physics, Keio University, Yokohama, Kanagawa 223-8522, Japan
Center for Spintronics Research Network, Keio University, Yokohama, Kanagawa 223-8522, Japan

Prof. Dr. T. Taniyama
Department of Physics, Nagoya University, Chikusa-ku, Nagoya, Aichi 464-8602, Japan

Prof. Dr. K. Hamaya
Center for Spintronics Research Network, Graduate School of Engineering Science, The University of Osaka, 1-3 Machikaneyama, Toyonaka, Osaka 560-8531, Japan
Spintronics Research Network Division, Institute for Open and Transdisciplinary Research Initiatives, The University of Osaka, 2-1 Yamadaoka, Suita, Osaka 565-0871, Japan
Email Address: hamaya.kohei.es@osaka-u.ac.jp

1 Experimental Section

Sample Fabrication, Structural Characterization and Magnetic Measurements

Epitaxial Co_2FeSi films were grown on $LiNbO_3$ 128° Y-cut (LN-128Y) substrates by molecular beam epitaxy (MBE). After loading the LN-128Y substrates into an MBE chamber, a heat treatment at 500 °C for 1 hour with a base pressure of $\sim 10^{-7}$ Pa was conducted [1]. As shown in the experimental results of the main text (Figure 2 and 4), the heat treatment at 500 °C does not deteriorate the piezoelectric properties of the LN-128Y substrates. After the heat treatment, two distinct symmetrical patterns are observed from *in-situ* reflection high-energy electron diffraction (RHEED) images [**Figure S1(c)**] [1]. After the substrates were cooled to 200 °C, a Cr insertion layer with a thickness of $\sim$10 nm was epitaxially grown [**Figure S1(b)**]. Subsequently, a Co_2FeSi layer with a thickness of $\sim$30 nm was grown by co-evaporating Co, Fe, and Si using Knudsen cells, where the supplied atomic composition ratio of Co:Fe:Si was 2:1:1 during the growth process [2, 3, 4]. The crystal structure was investigated by x-ray diffraction (XRD), high-angle annular dark-field scanning transmission electron microscopy (HAADF-STEM), and energy dispersive x-ray spectroscopy (EDX) measurements. Magnetic properties were measured by using a vibrating sample magnetometer in a physical property measurement system (Quantum Design).

Magnetization Dynamics Measurements

Magnetization dynamics of an epitaxial Co_2FeSi/LN-128Y heterostructure were investigated by ferromagnetic resonance (FMR) measurements with a vector network analyzer and a coplanar waveguide (CPW), where transmission parameter S_{21} was measured [5]. We fabricated a CPW antenna for spin-wave excitation and an electrode to apply E on a SiO_2/Si substrate, as shown in Figure 3(a). The CPW antenna and the electrode are composed of Ti(5 nm) and Au(300 nm). The width and the spacing of the waveguide are 100 μm and 50 μm, respectively. An insulating SiO_2 layer was deposited on the CPW by a magnetron sputtering system to avoid electrical contact between the Co_2FeSi layer and the CPW. To obtain good electrical contacts, a Au(100 nm)/Ti(3 nm) electrode was evaporated on the backside of the LN-128Y substrates by an electron beam deposition system, where the epitaxial Co_2FeSi layer was also utilized as a top electrode. The obtained S_{21} parameter was used to estimate the value of α.

Theoretical Calculations

First-principles density functional theory calculations were carried out using the Vienna Ab initio Simulation Package code [6, 7] with the generalized gradient approximation for the exchange and correlation term [8]. The projector augmented-wave pseudopotential was used to describe the behavior of core electrons [9]. We used 41×41×25 k-point mesh for wave vector integration in the first Brillouin zone and an on-site Coulomb interaction $U = 3.922$ eV for Fe atom [10]. We calculated three-types structural ordering, $L2_1$-Co_2FeSi ($D0_3$-type disordering : 0 %), $D0_3$-$(Co_{1.5}Fe_{0.5})(Co_{0.5}Fe_{0.5})Si$ ($D0_3$-type disordering : 50 %), and $D0_3$-(CoFe)CoSi ($D0_3$-type disordering : 100 %) to consider the effect of the $D0_3$-type disordering on α. The virtual crystal approximation was used to describe the $D0_3$-type disordering [11]. The values of α were calculated using torque correlation model by Kamberský [12]. The calculated values of α for cubic $L2_1$-Co_2FeSi, $D0_3$-$(Co_{1.5}Fe_{0.5})(Co_{0.5}Fe_{0.5})Si$, and $D0_3$-(CoFe)CoSi were 0.0012, 0.0029, and 0.028, respectively. Since the calculated α value for $D0_3$-(CoFe)CoSi ($D0_3$-type disordering : 100 %) was nearly one order of magnitude larger than the experimental value, and the behavior of α with respect to the lattice strain, $c'/(2\sqrt{2}a')$, was also quite different from the experiment data. In the main text, therefore, we compared the experimental data with two-types of our calculated results for $L2_1$-Co_2FeSi ($D0_3$-type disordering : 0 %) and $D0_3$-$(Co_{1.5}Fe_{0.5})(Co_{0.5}Fe_{0.5})Si$ ($D0_3$-type disordering : 50 %).

Statistics Analysis

Pre-processing of data: For all the experiments, data pre-processing with the help of machine learning and artificial intelligence methods was not used.

Data presentation: For magnetic measurements, the value of the magnetization was divided by the volume of the ferromagnetic Co_2FeSi layer. The thickness of the Co_2FeSi layer was measured form cross-sectional HAADF-STEM images and the size of the sample was measured by using ImageJ (open software).

Sample size for each statistical analysis: Sample sizes were determined based on experimental equipments. For the growth of epitaxial $Co_2FeSi/LiNbO_3$ multiferroic heterostructures, LN-128Y substrates with a size of 1 cm × 1 cm were used. XRD measurements were performed in a size of 1 cm × 0.7 cm. VSM and FMR measurements were performed in a size of 0.3 cm × 0.3 cm.

Statical methods: From the experimental data of FMR measurements, the values of α were estimated following the procedure described in the main text of Section 2.2. The standard deviations of α were shown as error bars in the main panel of Figure 4.

Software Used: For magnetic measurements, Kaleida Glaph (Synergy Software) was used to subtract the background data from the raw data and ImageJ (open software) was used to measure the area of the measurement sample. For FMR measurements, Igor Pro (WaveMetrics) was used to estimate the values of α.

2 Growth of Co_2FeSi films on LN-128Y

Representative RHEED images during the growth of a Co_2FeSi layer on LN-128Y substrates are shown in **Figure S1**. After the heat treatment at 500 °C for 1 hour, two distinct symmetrical patterns are observed from *in-situ* RHEED images, as shown in **Figure S1(c)** [1]. After the substrates were cooled to 200 °C, a Cr insertion layer with a thickness of ∼10 nm was grown by evaporating Cr using a Knudsen cell. After the growth, clear streak patterns due to good two-dimensional epitaxial growth are observed, as shown in **Figure S1(b)**. Subsequently, a Co_2FeSi layer with a thickness of ∼30 nm was grown at 200 °C by co-evaporating Co, Fe, and Si using Knudsen cells, where the supplied atomic composition ratio of Co:Fe:Si was 2:1:1 during the growth process [2, 3, 4]. After the growth, streak patterns due to epitaxial growth are observed, as shown in **Figure S1(a)**. To confirm the effect of an insertion of a Cr layer, we also use V as an insertion layer. As a result, although the surface flatness of a V insertion layer is not good compared with Cr, epitaxial growth of Co_2FeSi is realized, as shown in **Figure S1(d) and (e)**. By the way, when we directly grow Co_2FeSi on LN-128Y, only polycrystalline films are obtained as shown in **Figure S1(f)**.

3 Magnetic properties

Figure S2(a) shows a field-dependent magnetization (M-H curve) at 300 K in the in-plane configuration for an epitaxial Co_2FeSi/LN-128Y heterostructure in a high magnetic field region, where an external magnetic field was applied with an angle of 90° with respect to the LN[$2\overline{1}\overline{1}0$] direction (a red arrow in the inset figure). The saturation magnetization is ∼1235 emu/cc, almost equivalent to that for bulk [13] and thin-film [2, 3, 14] samples reported previously. In **Figure S2(b)**, we show normalized in-plane M-H curves in a low magnetic field region, where an external magnetic field was varied with an angle of 0°, 30°, 60°, and 90° with respect to the LN[$2\overline{1}\overline{1}0$] direction (see the inset figure). The epitaxial Co_2FeSi/LN-128Y heterostructure exhibits a strong in-plane uniaxial magnetic anisotropy and its easy axis is perpendicular to the LN[$2\overline{1}\overline{1}0$] direction. Similar strong in-plane uniaxial magnetic anisotropy was also observed for epitaxial Co_2FeSi/LN-128Y heterostructure with an insertion of a V layer and poly-crystalline Co_2FeSi/LN-128Y one (not shown here). Unlike poly-crystalline ferromagnet/LN-128Y structures [15, 16], the M-H curve for the epitaxial Co_2FeSi/LN-128Y heterostructure show a hysteresis behavior even for the LN[$2\overline{1}\overline{1}0$] direction [blue curve of Figure S2(b)], which may be attributed to the presence of two crystal domains in the film plane of the epitaxial Co_2FeSi layer shown in the main text of Figure 2(a).

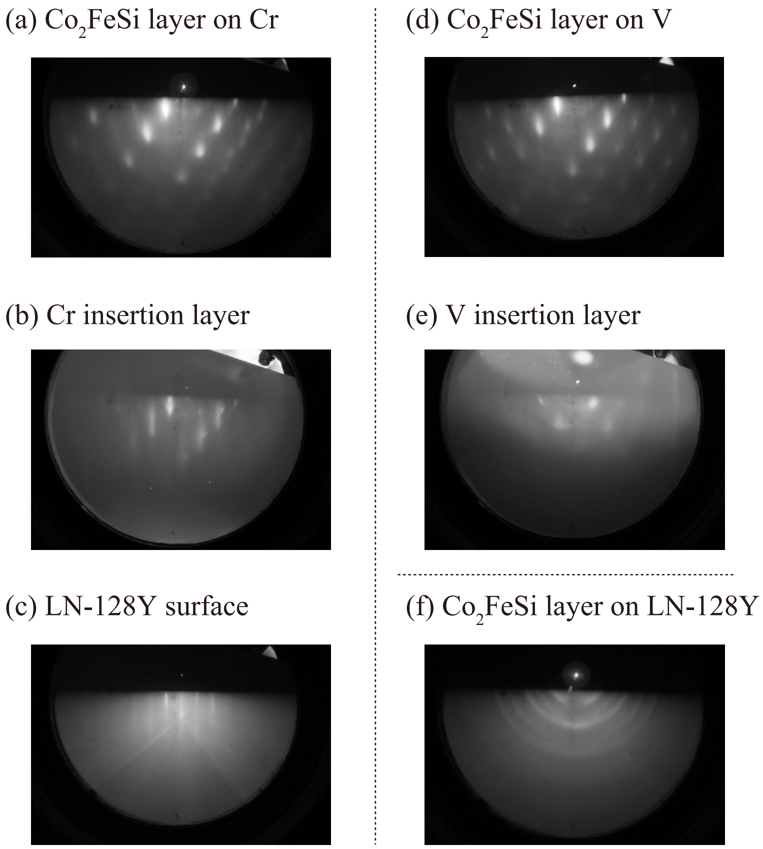

Figure S1: Representative RHEED images during the growth process of a Co_2FeSi layer on LN-128Y substrates with and without a bcc-metal insertion layer.

4 Theoretical calculations

Figure S3(a) and (b) show the density of states (DOS) for the majority and minority spins of $L2_1$-Co_2FeSi ($D0_3$-type disordering : 0 %) and $D0_3$-$(Co_{1.5}Fe_{0.5})(Co_{0.5}Fe_{0.5})Si$ ($D0_3$-type disordering : 50 %). The green line is the cubic-Co_2FeSi, and blue and red ones correspond to the cases of the compressive strain ($c'/(2\sqrt{2}a') = 1.716$) and the tensile strain ($c'/(2\sqrt{2}a') = 1.747$) in the Co_2FeSi layer, respectively. The enlarged views of the total DOS for the minority spins near E_F are shown in **Figure S3(c) and (d)**. For the $D0_3$-$(Co_{1.5}Fe_{0.5})(Co_{0.5}Fe_{0.5})Si$, although the width of a half-metallic gap near E_F is small compared with the $L2_1$-Co_2FeSi, large change cannot be seen in the DOS at E_F. For both $L2_1$-Co_2FeSi and $D0_3$-$(Co_{1.5}Fe_{0.5})(Co_{0.5}Fe_{0.5})Si$, the total DOS for the minority spin band decreases by applying a compressive strain.

Since the half-metallic gap of minority spin states of Co_2FeSi near the Fermi level consists mainly of Co d-orbitals, the projected DOS onto each d-orbitals of Co atom for $L2_1$-Co_2FeSi ($D0_3$-type disordering : 0 %) and $D0_3$-$(Co_{1.5}Fe_{0.5})(Co_{0.5}Fe_{0.5})Si$ ($D0_3$-type disordering : 50 %) are shown in **Figure S4(a)-(c)** and **Figure S4(d)-(f)**, respectively. It can be found that the in-plane orbitals of $d(xy, x^2 - y^2)$ are very sensitive to the distortion for Co_2FeSi with the $L2_1$ structure. On the other hand, out-of-plane orbitals $d(yz,zx)$ are more sensitive to the distortion of the $D0_3$ structure. These orbitals contribute to magnetization damping due to electron scattering, which changes the orbital quantum number through the spin-conserving terms in the matrix elements of the angular momentum operator L^- such as $\langle d(yz)|L^-|d(x^2 - y^2)\rangle$ and $\langle d(zx)|L^-|d(xy)\rangle$. We consider this to be the origin of the strain dependence of magnetization damping.

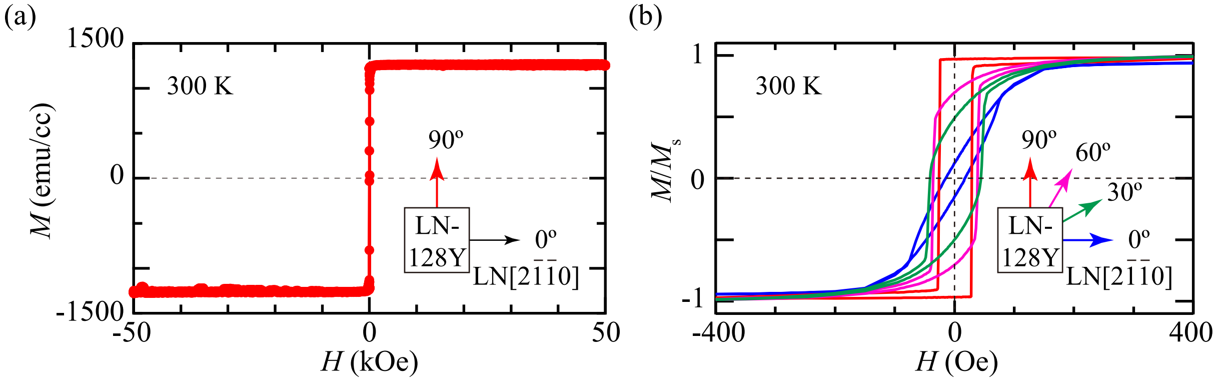

Figure S2: (a) High-field M-H curve at 300 K in the in-plane configuration for an epitaxial Co_2FeSi/LN-128Y multiferroic heterostructure measured with an angle of 90° with respect to the LN[$2\bar{1}\bar{1}0$] direction (red arrow). (b) Normalized low-field M-H curves measured in the in-plane configuration with an angle of 0° (blue), 30° (green), 60° (magenta), and 90° (red) with respect to the LN[$2\bar{1}\bar{1}0$] direction.

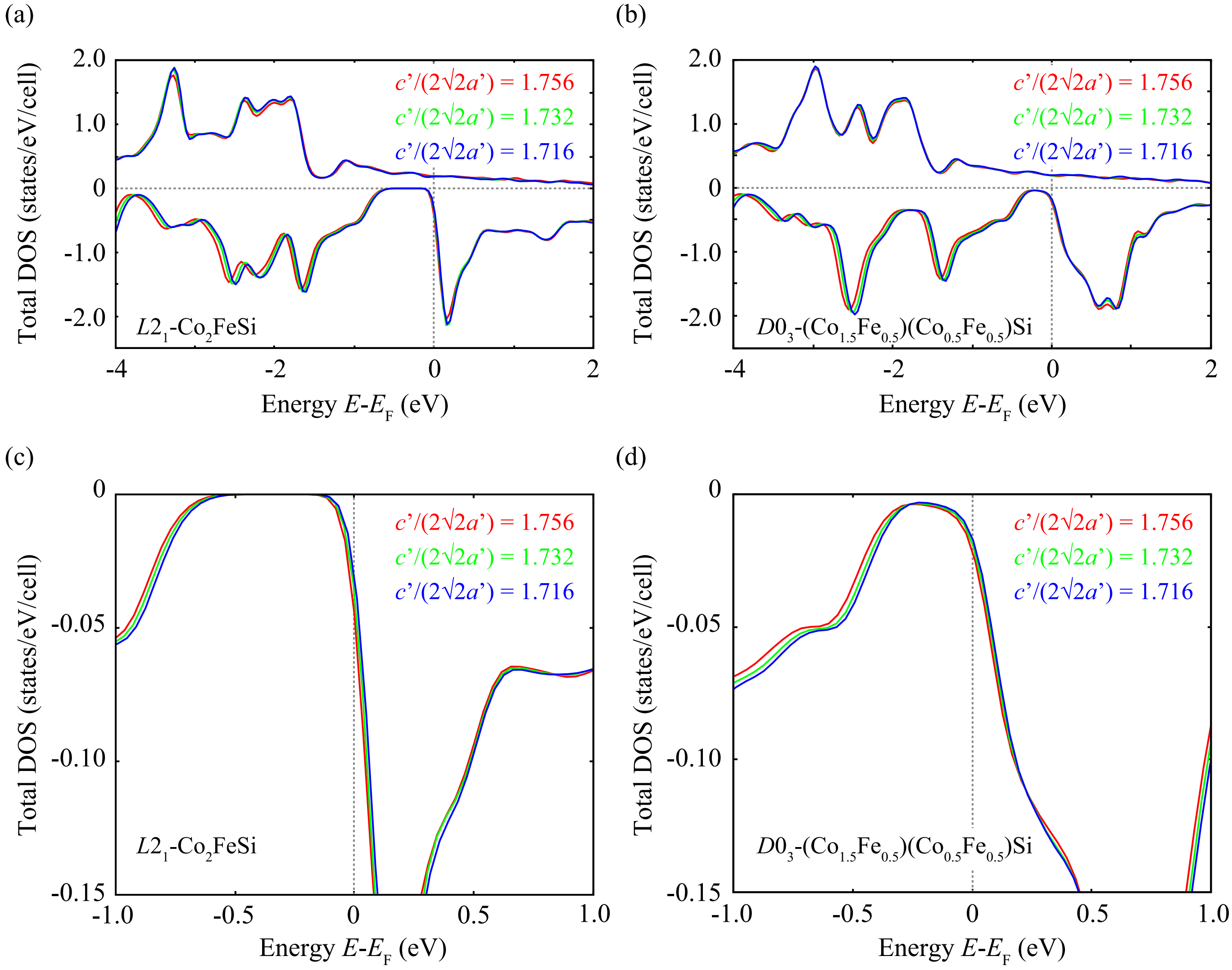

Figure S3: The total DOS for the majority and minority spins of (a) $L2_1$-Co_2FeSi (Co-Fe disordering : 0 %) and (b) $D0_3$-$(Co_{1.5}Fe_{0.5})(Co_{0.5}Fe_{0.5})Si$ (Co-Fe disordering : 50 %). [(c),(d)] Enlarged views of the total DOS for the minority spins near E_F of Figure S3(a) and (b).

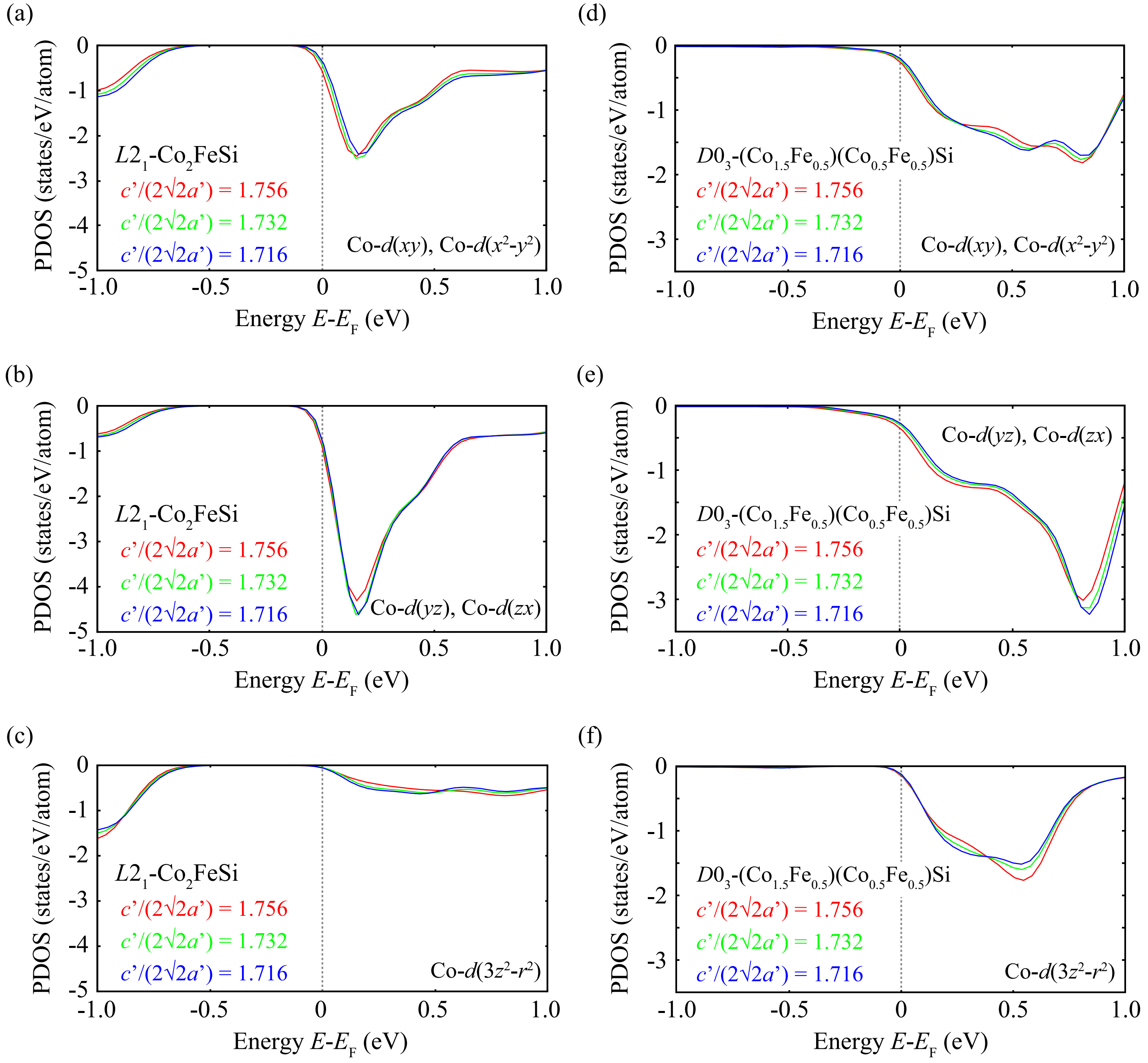

Figure S4: The projected DOS for the majority and minority spins of $L2_1$-Co_2FeSi (Co-Fe disordering : 0 %) for (a) Co-$d(xy, x^2 - y^2)$, (b) Co-$d(yz, zx)$ and (c) Co-$d(3z^2 - r^2)$, and $D0_3$-$(Co_{1.5}Fe_{0.5})(Co_{0.5}Fe_{0.5})Si$ (Co-Fe disordering : 50 %) for (d) Co-$d(xy, x^2 - y^2)$, (e) Co-$d(yz, zx)$, and (f) Co-$d(3z^2 - r^2)$.

References

[1] V. Polewczyk, M. Hehn, A. Hillion, S. Robert, P. Boulet, K. Dumesnil, ”Epitaxial growth of magnetostrictive $TbFe_2$ films on piezoelectric $LiNbO_3$,” *J. Phys.: Condens. Matter.* **2019**, *31*, 405801. https://doi.org/10.1088/1361-648X/ab27e6

[2] S. Yamada, K. Hamaya, K. Yamamoto, T. Murakami, K. Mibu, M. Miyao, ”Significant growth-temperature dependence of ferromagnetic properties for Co_2FeSi/Si(111) prepared by low-temperature molecular beam epitaxy,” *Appl. Phys. Lett.* **2010**, *96*, 082511. https://doi.org/10.1063/1.3330895

[3] S. Yamada, K. Tanikawa, S. Oki, M. Kawano, M. Miyao, K. Hamaya, ”Improvement of magnetic and structural stabilities in high-quality $Co_2FeSi_{1-x}Al_x$/Si heterointerfaces,” *Appl. Phys. Lett.* **2014**, *105*, 071601. https://doi.org/10.1063/1.4893608

[4] Y. Fujita, M. Yamada, M. Tsukahara, T. Oka, S. Yamada, T. Kanashima, K. Sawano, K. Hamaya, ”Spin Transport and Relaxation up to 250 K in Heavily Doped n-Ge Detected Using $Co_2FeAl_{0.5}Si_{0.5}$ Electrodes,” *Phys. Rev. Applied* **2017**, *8*, 014007. https://doi.org/10.1103/PhysRevApplied.8.014007

[5] T. Usami, M. Itoh, T. Taniyama, ”Temperature dependence of the effective Gilbert damping constant of FeRh thin films,” *AIP Advances* **2021**, *11*, 045302. https://doi.org/10.1063/5.0039577

[6] G. Kresse and J. Furthmüller, ”Efficiency of ab-initio total energy calculations for metals and semiconductors using a plane-wave basis set,” *Comput. Mater. Sci.* **1996**, *6*, 15. https://doi.org/10.1016/0927-0256(96)00008-0

[7] G. Kresse and J. Furthmüller, ”Efficient iterative schemes for *ab initio* total-energy calculations using a plane-wave basis set,” *Phys. Rev. B* **1996**, *54*, 11169. https://doi.org/10.1103/PhysRevB.54.11169

[8] J. P. Perdew, K. Burke, M. Ernzerhof, ”Generalized Gradient Approximation Made Simple,” *Phys. Rev. Lett.* **1996**, *77*, 3865. https://doi.org/10.1103/PhysRevLett.77.3865

[9] P. E. Blöchl, ”Projector augmented-wave method,” *Phys. Rev. B* **1994**, *50*, 17953. https://doi.org/10.1103/PhysRevB.50.17953

[10] K. Nawa, Y. Miura, ”Exploring half-metallic Co-based full Heusler alloys using a DFT+U method combined with linear response approach,” *RSC Adv.* **2020**, *10*, 44633. https://doi.org/10.1039/C9RA05212G

[11] C. Eckhardt, K. Hummer, G. Kresse, ”Indirect-to-direct gap transition in strained and unstrained Sn_xGe_{1-x} alloys,” *Phys. Rev. B* **2014**, *89*, 165201. https://doi.org/10.1103/PhysRevB.89.165201

[12] V. Kamberský, ”On ferromagnetic resonance damping in metals,” *Czech. J. Phys. B* **1976**, *26*, 1366. https://doi.org/10.1007/BF01587621

[13] S. Wurmehl, G. H. Fecher, H. C. Kandpal, V. Ksenofontov, C. Felser, H.-Ji Lin, J. Morais, ”Geometric, electronic, and magnetic structure of Co_2FeSi: Curie temperature and magnetic moment measurements and calculations,” *Phys. Rev. B* **2005**, *72*, 184434. https://doi.org/10.1103/PhysRevB.72.184434

[14] K. Kudo, Y. Hamazaki, S. Yamada, S. Abo, Y. Gohda, K. Hamaya, ”Great differences between low-temperature grown Co_2FeSi and Co_2MnSi films on single-crystalline oxides,” *ACS Appl. Electron. Mater.* **2019**, *1*, 2371. https://doi.org/10.1021/acsaelm.9b00546

[15] M. Ito, A. Yamaguchi, D. Oshima, T. Kato, M. Shima, K. Yamada, "Enhancement of spin-orbit torques by change in uniaxial in-plane magnetic anisotropy of Py/Pt bilayers on single crystal 128° Y-Cut $LiNbO_3$ substrate," *Appl. Phys. Lett.* **2021**, *119*, 152407. https://doi.org/10.1063/5.0063207

[16] M. Ito, S. Ono, H. Fukui, K. Kogirima, N. Maki, T. Hikage, T. Kato, T. Ohkochi, A. Yamaguchi, M. Shima, K. Yamada, "Uniaxial in-plane magnetic anisotropy mechanism in Ni, Fe, and Ni-Fe alloy films deposited on single crystal Y-cut 128° $LiNbO_3$ using magnetron sputtering," *J. Magn. Magn. Mater.* **2022**, *564*, 170177. https://doi.org/10.1016/j.jmmm.2022.170177
